# A rare convergence: Gangrenous bowel secondary to closed-loop obstruction with elevated urine amylase levels - A comprehensive case report

**DOI:** 10.51866/cr.624

**Published:** 2024-08-21

**Authors:** Hassan Ahmed Abrizan, Sani Mohamad Ikhwan, Wan Mokhter Wan Mokhzani, Hashim Merican Isa Siti Rahmah, Maya Mazuwin Yahya, Syed Abd Aziz Syed Hassan

**Affiliations:** 1 MD, MMed (Surgery), Department of Surgery, Hospital Universiti Sains Malaysia, Jln Raja Perempuan Zainab II, Kubang Kerian, Kota Bharu, Kelantan, Malaysia. Email: ikhwansani@yahoo.com.my; 2 MD, Department of Surgery, Hospital Universiti Sains Malaysia, Jln Raja Perempuan Zainab II, Kubang Kerian, Kota Bharu, Kelantan, Malaysia.; 3 MD, MMed (Surgery), Department of Surgery, Universiti Sains Malaysia, Jln Raja Perempuan Zainab II, Kubang Kerian, Kota Bharu, Kelantan, Malaysia.; 4 MD, MMed (Surgery), Department of Surgery, Universiti Sains Malaysia, Jln Raja Perempuan Zainab II, Kubang Kerian, Kota Bharu, Kelantan, Malaysia.; 5 MB BCh BAO, MMed (Surgery), Department of Surgery, Universiti Sains Malaysia, Jln Raja Perempuan Zainab II, Kubang Kerian, Kota Bharu, Kelantan, Malaysia.; 6 MBBS, MMed (Surgery), Department of Surgery, Universiti Sains Malaysia, Jln Raja Perempuan Zainab II, Kubang Kerian, Kota Bharu, Kelantan, Malaysia.

**Keywords:** Amylase, Hyperamylasaemia, Pancreatitis, Pancreas

## Abstract

Urine amylase levels are usually used to diagnose acute pancreatitis. However, there are reported cases where urine amylase levels are slightly increased in individuals without pancreatitis. Herein, we report the case of a young lady who presented with acute abdominal pain for 3 days. Her urine amylase level was 1717 U/L upon admission, and her condition was initially treated as acute pancreatitis. Unfortunately, the patient demonstrated abdominal guarding after 24 h; thus, urgent computed tomography (CT) was performed. CT revealed the presence of a dilated small bowel. She underwent emergency laparotomy, wherein a gangrenous small bowel with no evidence of saponification at the lesser sac was noted. Due to the non-specific nature of hyperamylasaemia, an alternative diagnosis other than acute pancreatitis should be considered if the clinical symptoms are not suggestive of pancreatitis or the condition worsens despite conservative management.

## Introduction

Amylase is a digestive enzyme with amylolytic properties that play a pivotal role in breaking down starch into smaller polysaccharides, ultimately yielding monosaccharides. Serum and urine amylase levels threefold higher than the normal limit are detected in approximately 75% of pancreatitis cases.^1^ Amylase is released into the urine for an extended duration, persisting for several days following the normalisation of serum amylase levels. This suggests that urinary amylase excretion could serve as a more dependable and sensitive marker for acute pancreatitis in numerous instances.^2^

## Case presentation

A 26-year-old woman presented with persistent abdominal pain of sudden onset, described as pricking in nature, with no aggravating factors. The pain was located in the epigastric and umbilical regions, radiating to the left and right hypochondria but not to the back. She had experienced five episodes of vomiting with food contents but no greenish discharge. There had been no bowel output for the past 2 days, and she passed flatus only the night before.

**Figure 1:**
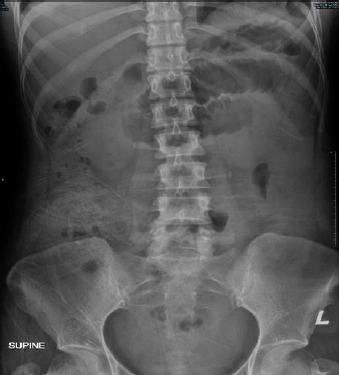
Abdominal radiography showed prominent small bowel loops.

Upon examination, the patient was alert, conscious, not tachypnoeic and lethargic. Her vital signs remained stable. Abdominal examination revealed soft distension with sluggish bowel sounds and no ascites. Blood investigation during admission showed a raised total white blood cell count but normal liver function and renal profile. The serum amylase level was measured at 70 U/L. Urine full and microscopic examination and urine pregnancy test results were negative, but a high urine amylase level of 1717 U/L was noted. Abdominal radiography revealed prominent small bowel loops (Figure 1). Ultrasound of the hepatobiliary system showed no sonographic features of acute pancreatitis or biliary calculus.

**Figure 2:**
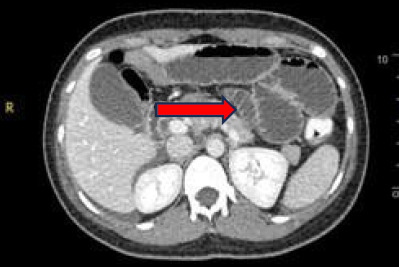
Computed tomography showed a dilated small bowel (arrow).

Contrast-enhanced computed tomography (CT) of the abdomen and pelvis revealed long- segment dilatation of the small bowel loops, measuring up to 3.6 cm in the widest diameter (Figure 2). There was an abrupt tapering at the left iliac fossa region with two adjacent transition points, giving the appearance of the double beak sign. CT showed no evidence of acute pancreatitis. The patient was then scheduled for emergency laparotomy.

**Figure 3:**
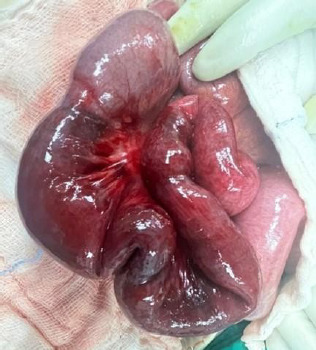
Congested and dusky part of the small bowel.

Intraoperatively, the small bowel was found to be dilated, with a dusky appearance and haemoserous ascitic fluid. A transition point was identified 100 cm from the duodenaljejunal junction, with a constricting point seen. A second constricting point was identified 20 cm distal to the first. The findings suggested a closed-loop obstruction due to an adhesion band (Figure 3). Segmental bowel resection with primary anastomosis was performed. Upon entry to the lesser sac, no saponification to indicate acute pancreatitis was observed.

Postoperatively, the patient recovered without any major complication and was discharged on the fifth day with oral antibiotics.

## Discussion

Acute pancreatitis typically manifests with constitutional symptoms, including nausea, vomiting, abdominal pain and disturbances in glucose or calcium levels, accompanied by radiological changes in the pancreas. Conversely, asymptomatic hyperamylasaemia is commonly associated with systemic diseases such as tumours, mumps and kidney failure or drug usage.^1^ Acute pancreatitis is a frequently encountered condition, carrying substantial risks of mortality and morbidity.^3^ According to the United Kingdom guidelines for the management of acute pancreatitis, the diagnosis of acute pancreatitis relies on the combination of clinical features (e.g. abdominal pain and vomiting) and an elevation in the plasma concentration of pancreatic enzymes.^3^

The assessment of an acute abdomen often relies on the measurement of total serum amylase due to its cost-effectiveness and technical simplicity, making it widely utilised. However, while total serum amylase is commonly employed, it lacks specificity as a diagnostic marker for acute pancreatitis. Estimates suggest a specificity approaching 95% when considering the threshold level to be more than three times the upper limit of the normal range.^4^

Hyperamylasaemia is not exclusive to pancreatic conditions and has been documented in various non-pancreatic disorders, such as mumps, parotitis, perforated peptic ulcer, perforated appendicitis, intestinal obstruction, mesenteric infarction, pulmonary embolism, pneumonia, myocardial infarction, lung cancer, breast cancer, lymphoma and several tubo-ovarian disorders.^3^

Lipase is recommended over amylase for diagnosing acute pancreatitis, as supported by various studies indicating its superior sensitivity and specificity (>95%) over total amylase in identifying pancreatic disease.^4^ Lipase has demonstrated higher diagnostic accuracy than pancreatic amylase.^5^ However, akin to pancreatic amylase, lipase is not exclusive to the pancreas, being present in the tongue, oesophagus, stomach, duodenum, small bowel, liver, lung and adipose tissue.^6^ Consequently, hyperlipasaemia has been associated with conditions such as cholecystitis, oesophagitis, peptic ulcer disease, enteritis, peritonitis and bowel obstruction and infarction.^7^ Official guidelines in the United Kingdom currently advise limiting the use of CT in diagnosing acute pancreatitis to cases where clinical and biochemical findings are inconclusive.^3^ The present case underscores the limitations of relying solely on amylase and lipase measurements and highlights the importance of emergency CT as a diagnostic adjunct in cases of acute abdominal pain.

## Conclusion

In conclusion, the present case shows the challenges in diagnosing acute abdominal conditions solely based on enzymatic markers. While amylase levels are used due to their simplicity and cost-effectiveness, healthcare providers should consider integrating clinical assessments with advanced imaging techniques early in the diagnostic pathway to enhance diagnostic accuracy and promote timely management of acute abdominal conditions such as pancreatitis or bowel obstruction. This strategy can help differentiate between conditions that mimic acute pancreatitis and those that require urgent surgical intervention.

## References

[1] Al-Johani WM (2023). Macroamylasemia as a rare cause of hyperamylasemia: a case report.. Korean J Fam Med..

[2] Judal H, Ganatra V, Choudhary PR (2022). Urinary amylase levels in the diagnosis of acute pancreatitis: a prospective case control study.. Int Surg J..

[3] Sinha S, Khan H, Timms PM, Olagbaiye OA (2010). Pancreatic-type hyperamylasemia and hyperlipasemia secondary to ruptured ovarian cyst: a case report and review of the literature.. J EmergMed..

[4] Matull WR, Pereira SP, Donohue JW (2006). Biochemical markers of acute pancreatitis.. J Clin Pathol..

[5] Yang RW Shao ZX, Chen YY, Yin Z, Wang WJ (2005). Lipase and pancreatic amylase activities in diagnosis of acute pancreatitis in patients with hyperamylasemia.. Hepatobiliary Pancreat Dis Int..

[6] Frank B, Gottlieb K (1999). Amylase normal, lipase elevated: is it pancreatitis? A case series and review of the literature.. Am J Gastroenterol..

[7] Chase CW, Barker DE, Russell WL, Burns RP (1996). Serum amylase and lipase in the evaluation of acute abdominal pain.. Am Surg..

